# The difficulties of recanalization in chronic superior cava vein obstruction by penetrating diagnostic catheter, PTA, and stenting: A case report

**DOI:** 10.1016/j.radcr.2024.02.081

**Published:** 2024-03-21

**Authors:** Jacub Pandelaki, Antono Sutandar, Elisa Feriyanti Pakpahan, Anna Celesta Nastasia Nalley, Rahmad Rizky Aprial, Prijo Sidipratomo

**Affiliations:** a Department of Radiology, Dr. Cipto Mangunkusumo National General Hospital - Faculty of Medicine, Universitas Indonesia, Jakarta, Indonesia; b Department of Radiology, Siloam Kebon Jeruk General Hospital, Jakarta, Indonesia; c Department of Radiology, Siloam Kupang General Hospital, Kupang, East Nusa Tenggara, Indonesia; d Department of Radiology, Hadji Boejasin Regional General Hospital, Pelaihari, South Kalimantan, Indonesia

**Keywords:** Superior vena cava obstruction, Diagnostic catheter, Percutaneous transluminal angioplasty, Stenting, Recanalization, Thrombectomy

## Abstract

Superior vena cava obstruction is caused by a blockage in its blood flow; one of its few causes can be device related. This case follows a patient presented with superior vena cava obstruction following a septal cardiac implant. Endovascular intervention has been associated with more rapid, complete symptom relief and lower complication rates. The use of stenting as first-line therapy has gathered popularity to become standard practice in the past 2 decades. This paper illustrates a successful recanalization with penetrating diagnostic catheter, followed with percutaneous transluminal angioplasty stenting in order to preserve the patency superior vena cava.

## Introduction

Superior vena cava (SVC) syndrome is a collection of symptoms caused by partial or total SVC obstruction (SVCO). Mediastinal malignancy (bronchogenic, lymphoma, and metastatic) is the most prevalent aetiology, followed by infectious and intragenic (pacemakers and invasive access) injuries [1,2]. To confirm the diagnosis, information regarding the severity of the central venous occlusion and extensive venous collateralization can be obtained by performing contrast-enhanced Computed Tomography (CECT), Magnetic Resonance Imaging, or conventional venograms [2]. The goal of the treatment is to reduce venous pressure through either medical management or surgery (open or endovascular) [1]. The selection for the management of endovascular treatment is mechanical thrombectomy, thrombolysis, and venous stenting [3]. Several techniques, including the use of stiff wires and straight catheters, can be utilized to recanalize the vein when there is total occlusion [4]. In this case report, diagnostic catheter manipulation successfully recanalized the superior vena cava obstruction, followed by percutaneous transluminal angioplasty (PTA) and stenting, with good clinical outcomes.

## Case illustration

**Fig. 1:**
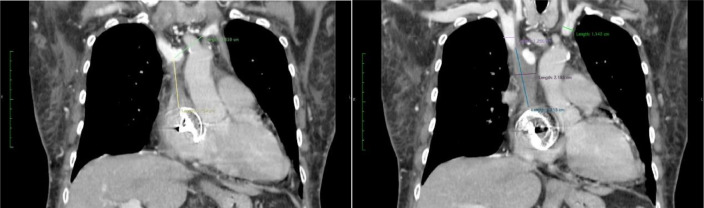
Thorax CECT shows a thrombus in the SVC with an Amplatzer Atrial Septal Occluder installed.

A 71-year-old woman was admitted with face, neck, and right hand swelling 7 days ago. Four years ago, the patient underwent an Amplatzer Atrial Septal Occluder^TM^ (Abbott Vascular, Lakeside Drive, Santa Clara, California) placement. Since, 1 year ago, the patient has routinely undergone hemodialysis due to chronic kidney disease. A venous catheter, exchanged every month, was placed alternately between the right and left jugular veins until an arteriovenous fistula in the right arm was created 2 months before the symptoms. Upon admission, thorax CECT examination showed a thrombus at the SVC with the presence of the septal occluder (Fig. 1).

Under general anesthesia, 5000 IU intravenous bolus of heparin was administered at the start of the procedure. The right common femoral vein was canulated using a 6-Fr. introducer sheath and a 5-Fr. Glidecath angled tapered angiographic catheter (Terumo Medical Canada Ltd., Keele Street, Vaughan, Canada), advanced until it reached the cavoatrial junction. Total occlusion of the superior vena cava was revealed upon venographic evaluation.

**Fig. 2:**
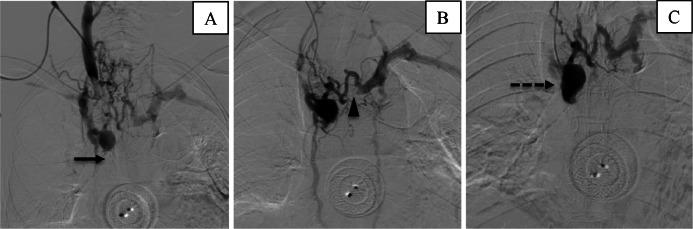
Venography shows occlusions in (A) the SVC (arrow) and (B) the left innominate vein (arrowhead) suggesting a type-D Stanford classification system for SVC Syndrome [9]. (C) Visualization of beak-like appearance (dashed arrow) after attempting recanalization.

Aspiration thrombectomy was performed using Indigo CAT8^TM^ Mechanical Thrombectomy Catheter and Indigo Separator^TM^ 8. (Penumbra Inc., Alameda, California, US) but was unable to penetrate the SVCO due to the thrombus location at the cavoatrial junction, which does not allow enough room for maneuver. A 6-Fr. radial introducer sheath was then utilized to establish right external jugular venous access. Venography revealed total occlusions of the distal superior vena cava and the left distal innominate vein, stenosis at the cavojugular junction, and collaterization via azygos-hemiazygos veins. A 5-Fr. vertebral catheter (Terumo Corporation, Hatagaya, Shibuya-Ku, Tokyo, Japan) was used, however, unsuccessful in penetrating the thrombus. Finecross MG 1.8-2.6-Fr. microcatheter (Terumo Corporation, Hatagaya, Shibuya-Ku, Tokyo, Japan) in combination with 0.014" chronic total occlusion (CTO) Pilot Wire (Abbott Vascular, Lakeside Drive, Santa Clara, California) was then inserted coaxially through a 5-Fr. Glidecath. Penetration of the thrombus was achieved by the CTO pilot wire, resulting in a beak-like dent of the thrombus on follow-up venography. Subsequently, a 5-Fr. Judkins-Right 4.0 catheter (Merit Medical Systems inc., South Jordan, Utah, US) and 0.035" Conquest Pro guide wire (Asahi Intecc Co., LTD, Akatsuki-Cho, Seto, Aichi, Japan) was inserted and was able to successfully penetrate the thrombus and into the right atrium (Fig. 2).

**Fig. 3:**
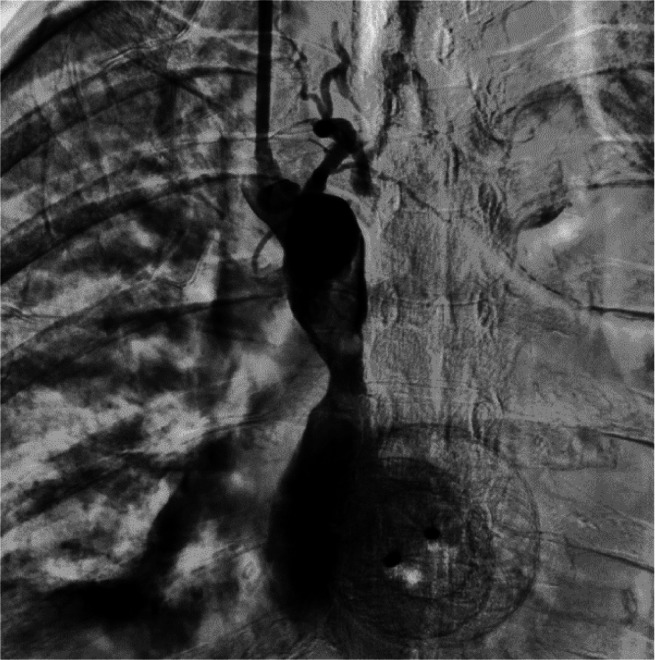
Venography after penetrating diagnostic catheter with subsequent PTA.

To allow the insertion and inflation (inflated twice at the stenotic site for 60 seconds each at pressures of 10 and 12 atm) of 8 mm x 80 mm PTA balloon dilatation Millou catheter (Cnovate Medical BV, Hogenbrinkerweg, Houvelaken, The Netherlands), the 6-Fr. jugular access sheath was replaced with an 8-Fr. introducer sheath. Venography performed after the balloon angioplasty procedure revealed no residual stenosis of the superior vena cava (Fig. 3). Due to stent unavailability at that moment, the procedure was stopped and a venous catheter was placed to preserve right jugular vein access. The right jugular introducer sheath was removed, leaving the guide wire in place to guide the insertion of 11.5-Fr. Triple-lumen non-tunneled Tri-flow™ catheter (Medical Component Inc., Delp Drive, Harleysville, US) with the help of a dilator. The tip of the venous catheter was placed in the distal superior vena cava. Throughout the whole procedure, which lasted around 3 hours, 2 bolus doses of 3000 IU heparin were given with a total intraprocedural heparin dose of 11,000 IU including the dose given at the start of the procedure. After the procedure, a maintenance heparin dose of 100 IU hourly was ordered for 24 hours, and an activated clotting time (ACT) value of 200-300s was maintained.

**Fig. 4:**
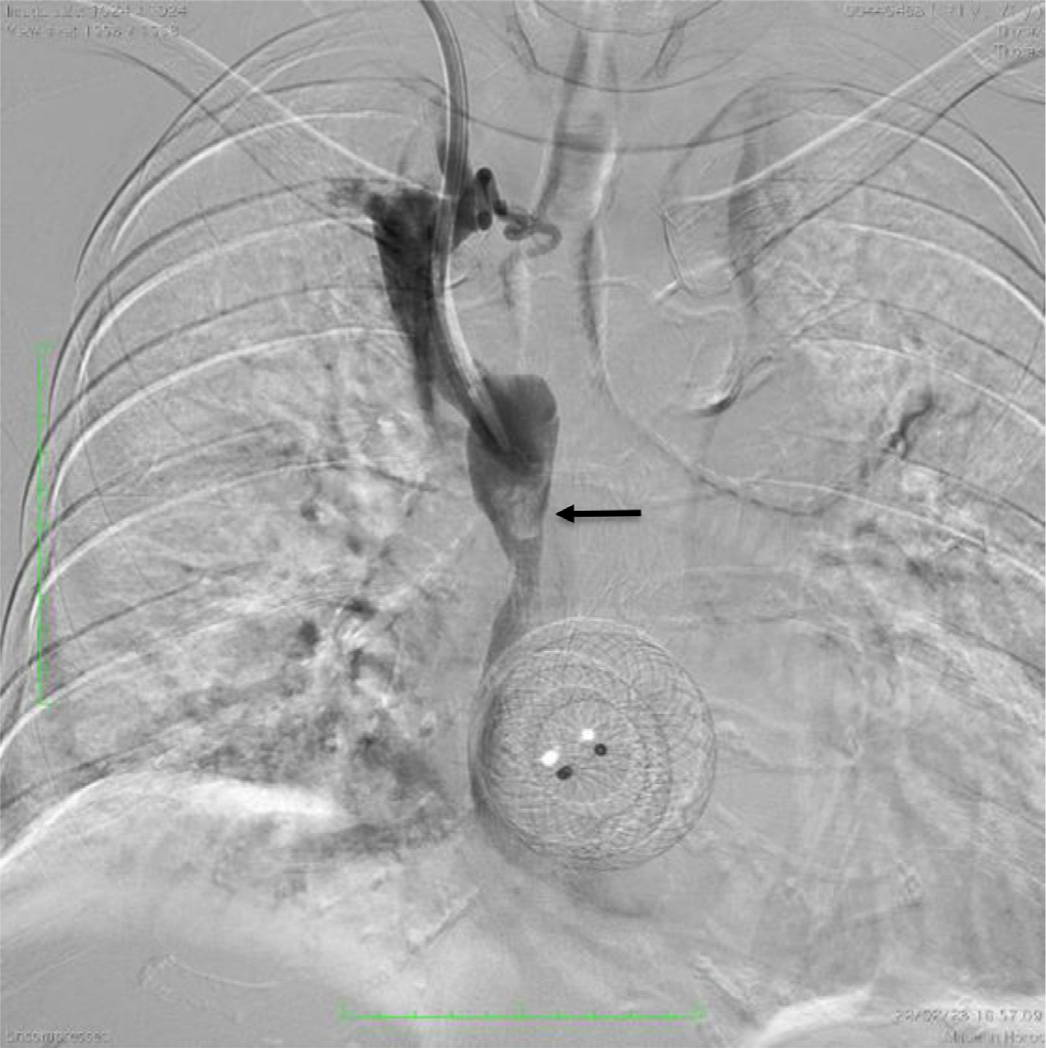
Appearance of a thrombus (arrow) in the SVC, prior to the second procedure.

**Fig. 5:**
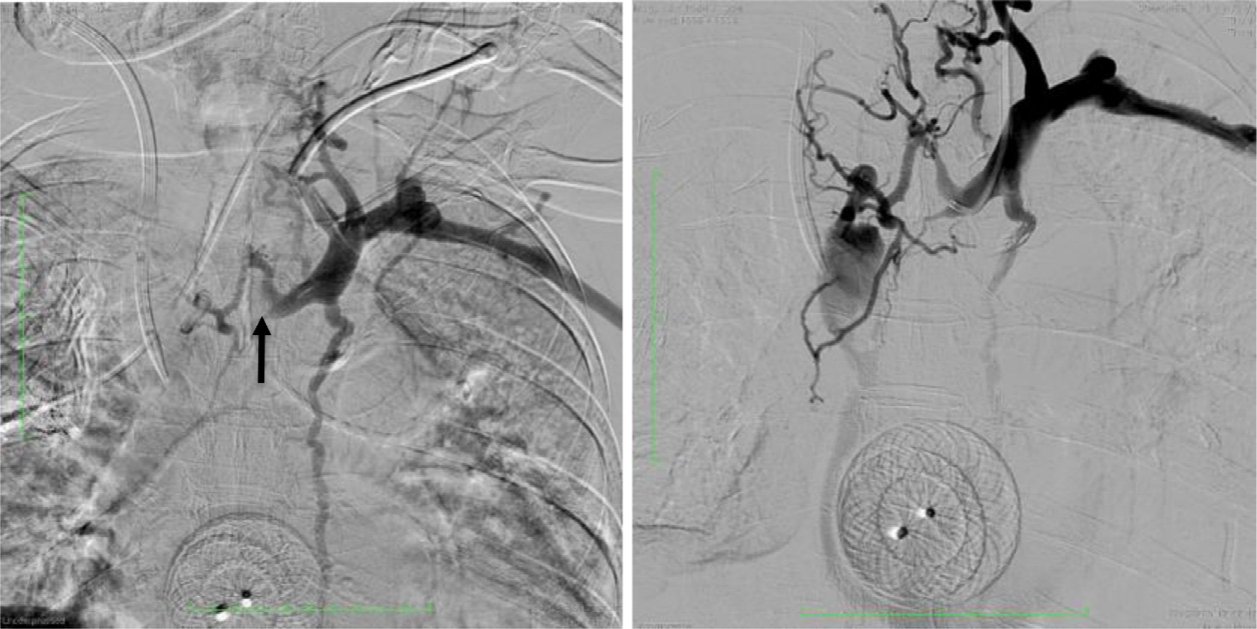
Occlusion in the left innominate vein (arrow) with collaterals to the hemiazygos vein.

The swelling of the patient's right arm was significantly reduced 1 week after the procedure was performed, but no discernible improvement was observed of the patient's left arm. Symptoms especially the swelling of the patient's right arm resurfaced after 2 weeks and venography through the venous catheter revealed thrombus in the SVC, as well as an occlusion of the left innominate vein (Fig. 4). Recanalizations of the occluded left innominate vein (Fig. 5) utilizing JR 4 from the left jugular and right femoral veins were tried. However, it is unfeasible due to the impenetrable thrombus and to avoid complications such as rupture with the risk of mediastinal bleeding.

**Fig. 6:**
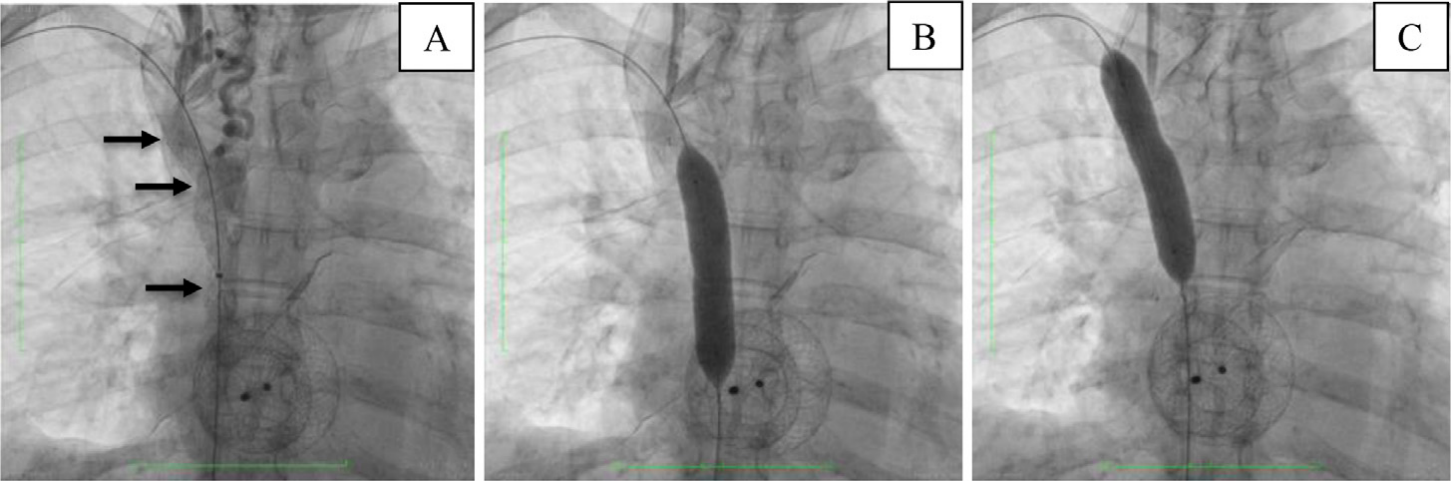
Deployment of (A) stent (arrow) followed by PTA in the (B) proximal and (C) distal SVC.

**Fig. 7:**
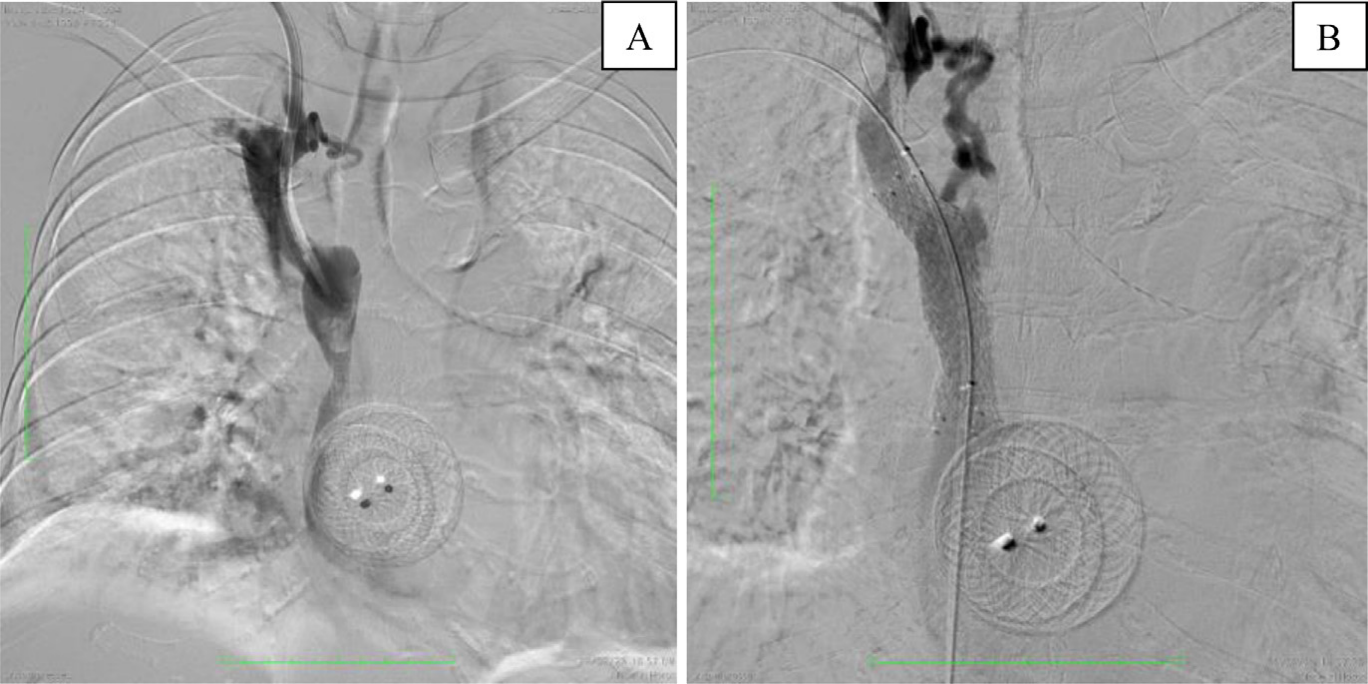
Venography (A) before and (B) after the stent placement and PTA.

Deployment of a 14 × 60 mm Epic Self-Expandable Stent (Boston Scientific Corporation, Malborough, MA, US) on the SVC stenosis via right femoral access, the venous catheter on the right jugular vein was temporarily partially retracted. Upon deployment, the stent was not able to fully expand (Fig. 6). Therefore, a 14 × 60 mm Armada balloon dilatation catheter (Abbot Vascular, Lakeside Drive, Santa Clara, CA, US), inflated 4 times at 4 atm for 60 seconds, was used to assist the stent expansion (Fig. 6). Venography evaluation after the procedure confirmed no further stenosis of the SVC and the venous catheter was repositioned (Fig. 7). Swelling of the right hand was markedly reduced with no recurrence after a 2-month follow-up.

The venous catheter is withdrawn partially until proximal above the PTA balloon and stent planning position. Deployment of a 14 × 60 mm Epic Self-Expandable Stent continued with the installation of 14 × 60 mm balloon dilatation catheter to assess the SVC that did not open optimally. After the surgery, the venous catheter tip is reinserted into the stent at the level of the cavojugular junction.

## Technique

A 5000 IU IV bolus of heparin was administered prior to the intervention, and another 3000 IU bolus was administered after the ACT test, which was conducted an hour into the intervention and obtained 116s. A repeat ACT was done before the insertion of the diagnostic catheter, and the results were 201 s. A stiff and rigid catheter might be combined with the CTO pilot wire to help penetrate the total occlusion. The catheter should be maneuvered and pushed with the curved-blunt side to avoid SVC dissection. In this case, twisting the JR cath and a 0.014" CTO pilot wire with a blunt curved approach allowed it to pass through the occlusion and enter the atrium. Heparin 1000 IU/hour maintenance over the next 24 hours, followed by a test 2 hours later with an ACT maintained in the 200-300s. Maintaining ACT above 200s is to avoid clotting by the thrombus.

## Discussion

SVC Syndrome is caused by a blockage in the SVC's blood flow. It is typically a complication of a malignancy and can also be caused by thrombosis from disrupted blood flow or blood coagulation disorder [5]. Patients with SVC syndrome should get a multidisciplinary care, with options for treatment including catheter-based thrombectomy/thrombolysis/PTA and Stent (endovascular intervention), radiation therapy with or without chemotherapy (in malignancy associated SVCO), and surgical bypass [5]. Endovascular intervention has been associated with more rapid, complete symptom relief and lower complication rates. The use of stenting as first-line therapy has gathered popularity to become standard practice in the past two decades [5,6].

Device-related SVCO has been on the rise recently, accounting for approximately 30% of SVCO. The primary cause are chronic mechanical irritation and reaction to foreign body [7]. We were presented a patient with SVCO following an atrial septal occluder implant and repetitive venous catheter placement. The presence of implant in ASD and chronic mechanical irritation from the venous catheter can cause interruption of the flow from the SVC to the atrium, causing thrombosis development [5]. The findings in this study are in accordance with the Type-IV SVCO, or the Stanford Type-B. Bilateral ``kissing'' or Y-shape stenting might be used in the case of occlusion extending to the brachiocephalic or subclavian veins (Type-IV), but stent extension only to one brachiocephalic vein is sufficient for symptomatic relief, with safer and easier technique [6].

After twice failure thrombectomy attempt using Indigo CAT8^Ⓡ^ from right femoral access, and 0.014” CTO pilot wire from right jugular access, ended with successful thrombectomy using Judkins-Right 4.0 6Fr. This successful thrombectomy with rigid catheter also reported by Kolbel et al in chronic occlusion of iliac vein and IVC. A venous catheter then installed with the tip at the distal of SVC to secure access. At this point, a stent should be installed to maintain SVC patency [8]. It was not done in our study due to the unavailability of the stent at that time.

## Conclusion

This report depicted a successful recanalization of SVC using a penetrating diagnostic catheter, PTA, and stenting. According to the literature, in a symptomatic SVCO, stenting must be performed in order to preserve the patency superior vena cava. Further studies are still needed for this and other types of SVC obstructions.

## Patient consent

The authors have obtained consent from the patient for their data, including their medical history and imaging studies, to be published in this case report.
